# Non-Traumatic Lower-Limb Amputations: Outcome, Sex-Differences, Comorbidity Patterns and Temporal Trends from 2006 to 2022

**DOI:** 10.3390/jcm14124030

**Published:** 2025-06-06

**Authors:** Susanne Kaser, Bernhard Radlinger, Jana Blasinger, Nicolas Koellenberger, Verena Streitberger, Lena Kopp, Elena Bifano, Faisal Aziz, Harald Sourij, Georg Goebel, Josef Klocker

**Affiliations:** 1 Department for Internal Medicine I, Medical University Innsbruck, 6020 Innsbruck, Austria; bernhard.radlinger@i-med.ac.at (B.R.); nicolas.koellenberger@student.i-med.ac.at (N.K.); verena.streitberger@student.i-med.ac.at (V.S.); 2 Department of Vascular Surgery, Medical University of Innsbruck, 6020 Innsbruck, Austria; jana.blasinger@student.i-med.ac.at (J.B.); lena.kopp@student.i-med.ac.at (L.K.); elenabifano99@gmail.com (E.B.); josef.klocker@i-med.ac.at (J.K.); 3 Division of Endocrinology and Diabetology, Medical University of Graz, 8036 Graz, Austria; faisal.aziz@medunigraz.at (F.A.); ha.sourij@medunigraz.at (H.S.); 4 Institute of Clinical Epidemiology, Public Health, Health Economics, Medical Statistics and Informatics, Medical University of Innsbruck, 6020 Innsbruck, Austria; georg.goebel@i-med.ac.at

**Keywords:** diabetic foot syndrome, peripheral artery disease, lower-limb amputation, sex difference, comorbidities, mortality

## Abstract

**Background/Objectives:** Non-traumatic lower-limb amputations are associated with high mortality and a dramatic loss of quality of life. Peripheral artery disease and diabetes are the most common causes of non-traumatic lower-limb amputations. The aim of the study was to assess temporal trends in mortality, comorbidities and sex differences in patients with non-traumatic lower-limb amputations. **Methods**: A total of 1107 patients who underwent lower-limb amputation for non-traumatic causes at the Medical University of Innsbruck between 2006 and 2022 were reviewed and analyzed. Temporal trends in mortality, sex-differences in outcomes and the comorbidity spectrum were assessed. **Results**: In hospitals, 30-day and 1-year mortality has remained high from 2006 to 2022 (4.14%; 16.2%; 23.2%) with chronic kidney disease, heart failure and major amputations as predictors of 1-year mortality. Diabetes, peripheral artery disease and cerebrovascular disease were the most common causes of death in females, and liver disease, renal disease and myocardial infarction in males, respectively. Overall comorbidity frequency was high, with there being even increasing rates of coronary heart disease, atrial fibrillation and chronic pulmonary disease during the study period. Age at first amputation was significantly higher in women than in men (78.9 vs. 68.1 years). Median age increased and median LDL-cholesterol decreased in males but not in females during the time period. Major amputations were performed more frequently in females than in males as the first surgical intervention. **Conclusions**: Mortality and morbidity are high in patients with non-traumatic limb amputation. Our data underline the need of intensified risk-factor management with lower limb amputation, especially in females.

## 1. Introduction

Non-traumatic lower-limb amputation is associated with a dramatic loss of quality of life and high cardiovascular mortality in affected patients [1,2,3,4]. Peripheral artery disease (PAD) and diabetic foot syndrome (DFS) are the most frequent causes of non-traumatic lower-limb amputation [1]. Recent studies by Gyldenkerne and colleagues [5] found decreasing rates of lower-limb amputations only in patients with diabetes but not in the general population, suggesting improved management in people with diabetes but insufficient advances in prevention and therapy of peripheral artery disease. It is noteworthy that, in patients with diabetes, decreased amputation rates were reported for females only, while the opposite was found for males [6]. In Austria, where the present study was conducted, the prevalence of major amputations in patients with diabetes remained stable between 2014 und 2017 and was higher in men than in females [7]. While some data are available on lower-limb amputations in patients with diabetes, far less information is available on the general temporal trends of non-traumatic lower-limb amputations and especially on minor amputations.

Both underlying disorders, diabetes and peripheral artery disease, are not only characterized by increasing prevalence but also by marked sex differences in pathophysiology and clinical course. In type 2 diabetes, prevalence and absolute cardiovascular risk is higher in males than in females [8,9,10,11,12]; however, the relative risk for cardiovascular disease and mortality is greater in females [8].

Peripheral artery disease is more prevalent in females than in males; however, amputation rates were reported to be more common in males than in females [13,14]. While the sex-specific effects of traditional risk factors such as smoking [15,16,17], diabetes [18,19], hypertension [20,21], chronic kidney disease [22,23] and LDL-C [14,24] in the pathophysiology of peripheral artery disease have been defined in detail, less is known on sex differences in relation to the comorbidity spectrum, risk factor management and outcome in patients with lower-limb amputation due to PAD and/or DFS. Only very recently, the American Heart Association called for further research to overcome sex-based differences and disparities in peripheral vascular disease [25].

Here we set out to study sex differences and temporal trends in mortality, comorbidities and risk factors in patients who had undergone non-traumatic lower-limb amputation at a University Center Hospital in Austria between 2006 and 2022.

## 2. Methods

### 2.1. Study Population and Outcome

Medical records from patients who underwent non-traumatic lower-limb amputations between 2006 and 2022 at the Department of Vascular Surgery at the Medical University of Innsbruck, Austria were analyzed. Patients with a lower-limb amputation due to oncologic indication or implantation-related infection or trauma were excluded from this study. Indications for lower-limb amputations were complications of peripheral artery disease including arterial embolism and diabetic foot syndrome with neuropathic ulcers and refractory osteomyelitis.

Diagnoses, medications and information on surgical procedures were obtained from the medical reports. All diagnoses and comorbidities used for analysis were based on clinical and laboratory findings, except causes of death, which were based on ICD codes. Laboratory values were considered as eligible for analysis when obtained within a maximum of 3 months before surgical intervention. Presurgical body weight and BMI were used for analysis. Data on vital status were available from 1087 patients until the end of 2023. Causes of death were obtained from Statistics Austria and were classified according to the Charlson Comorbidity category [26]. In patients with multiple amputations, data from the first amputation were used for analysis.

For temporal trend analysis, the study period was divided into 4 subperiods. Data were analyzed in total and separately for the time periods 2006–2010 (period 1 (P1)), 2011–2015 (period 2 (P2)), 2016–2019 (period 3 (P3)) and for the SARS-CoV2 pandemic years 2020–2022 (period 4 (P4)). The SARS-CoV2 pandemic years were analyzed separately due to partly limited access to health care during this period.

Minor lower-limb amputations were defined as amputations distal to the ankle joint, while major amputations were defined as amputations proximal to or through the ankle joint.

The study was approved by the local ethics committee of the Medical University of Innsbruck (1108/2023).

### 2.2. Statistics

For statistical analysis R (Version 4.4.1) [27] was used together with RStudio (Version 3.6-4.) [28]. Survival data were retrieved from Statistics Austria, the national registry which holds official records of the survival data of Austrian citizens including ICD-10-coded causes of deaths.

The compareGroups R package was used for descriptive statistics [29]. If not otherwise specified, the Shapiro–Wilks test was used to test for normality and either perform parametric or non-parametric tests. For all used numerical variables, distribution was non-normal, so the Kruskal–Wallis Test was used for group-wise comparison. For categorical variables the Chi-Squared test was used, with the exception of cause-of-death analysis, where the Permutation Chi-Squared test was used due to the low number of expected frequencies. Trend testing was used in analysis for time-trends with Spearman’s rank correlation for numerical data and with the Mantel–Haenszel test for categorial data. All-*p*-values in the tables were adjusted for multiple testing using the Benjamini–Hochberg adjustment.

Survival analysis was conducted using the survival R package [30]. The data were right-censored with 31 December 2023 as the censoring date, due to survival data from Statistics Austria being available in yearly intervals. For univariate comparisons of the survival data, the log-rank test was used and Cox regression analysis was performed for multivariate data.

A logistic regression model was fitted for the survival data 12 months after the initial surgery using a binomial generalized linear model.

A *p*-value below 0.05 was considered as statistically significant. No corrections for F1-inflation were applied due to the explorative character of the study.

The ICD-10 codes were categorized into 17 categories based on the Charlson comorbidity index using the icd R package [31].

## 3. Results

### 3.1. Baseline Characteristics and Comorbidities

In total, 1107 patients were included in the study (Table 1). A minor amputation was performed as the first amputation in 752 patients (67.9%), while in 355 patients (32.1%) the first amputation was a major amputation. At least one re-amputation was performed in 51.3% of patients with minor amputations (minor re-amputation: 44.9%, major re-amputation: 17.2%) and in 24.5% of patients with major amputations (minor-re-amputations 3.94%, major re-amputations: 21.4%). The ratio of minor to major amputations remained stable during the observation period (Table 1).

The median age of the patient at the time of first amputation increased from 72.0 years to 75.3 years from P1 to P4, while the median BMI remained stable.

In total, 68.8% of patients were male with a trend towards an even higher male ratio until the end of the observation period.

Fifty-five percent of patients had diabetes with a slight but not significant trend towards lower rates during the observation time.

The median LDL-C levels significantly declined from 87.5 mg/dL in P1 to 74 mg/dL in P4, and the number of current smokers decreased during the observation period; in parallel statin use increased over time. In contrast to other cardiovascular risk factors, the prevalence of hypertension significantly increased with time affecting 86.9% in P4.

The frequencies of coronary heart disease, atrial fibrillation and chronic pulmonary disease increased significantly, while the prevalence of heart failure remained stable during the observation period. The rate of chronic kidney disease did not change during the observation period.

### 3.2. Survival Analysis and Causes of Death

**Figure 1:**
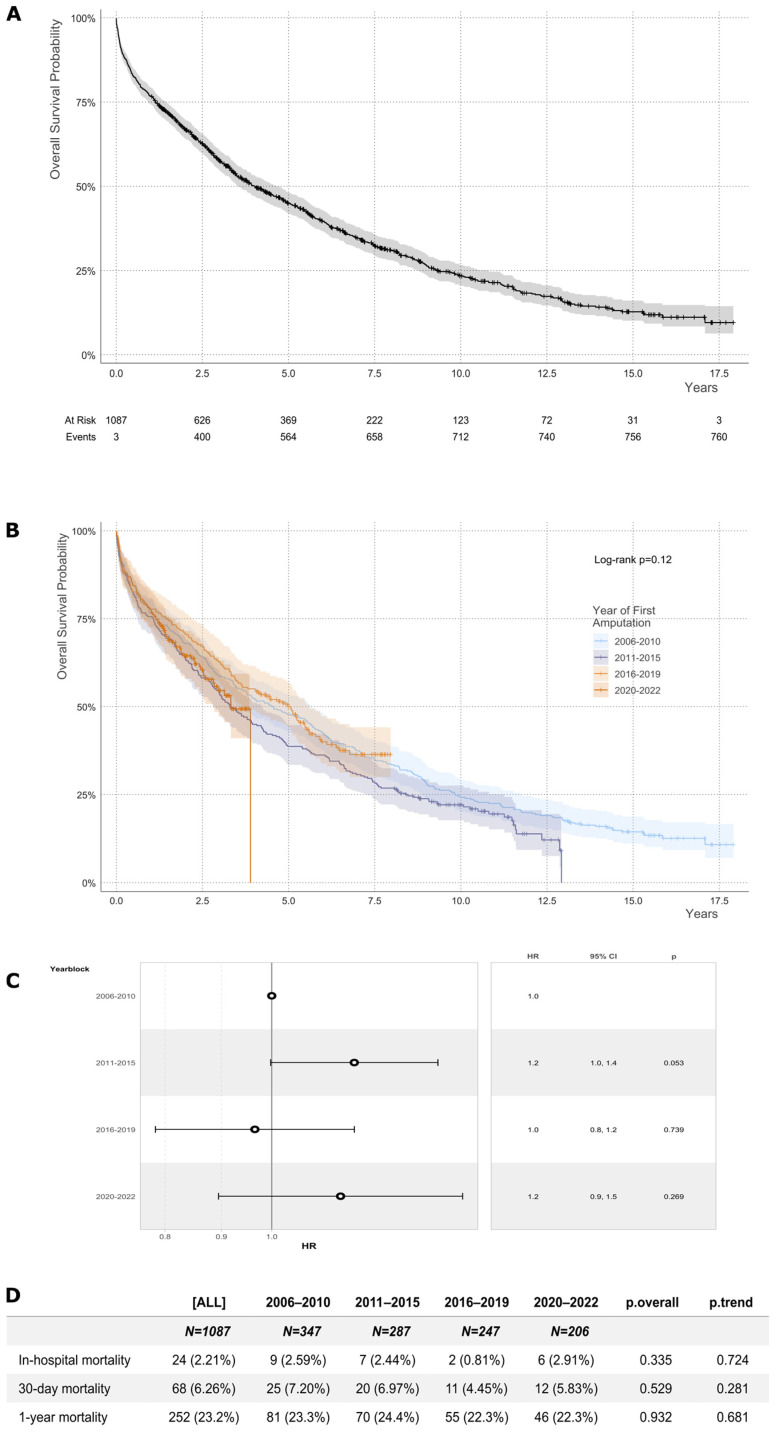
Overall survival probability. (**A**) The overall survival probability of all patients as estimated from the first amputation is shown. The shaded areas indicate the 95% confidence intervals. Censored data points are marked by ticks. The end of patient inclusion was 31 December 2022. The end of the follow-up of survival data was 31 December 2023. (**B**) Overall survival probability in four different time periods are shown. The log-rank test *p*-value is indicated for statistical comparison between groups. (**C**) The hazard ratios and confidence intervals for death in the different periods 1 to 4 are shown. (**D**) In-hospital mortality was calculated using demission dates. Figures for 30-day and 1-year mortality were calculated as death from the date of surgery, respectively.

The vital status was available for 1087 patients (98.2%). The median survival time was 4.03 years after the first amputation (Figure 1A). The overall survival did not significantly change over time (Figure 1B,C). The overall in-hospital mortality, 30-day mortality and 1-year mortality were 4.14%, 6.26% and 23.2%, respectively (Figure 1D).

**Figure 2:**
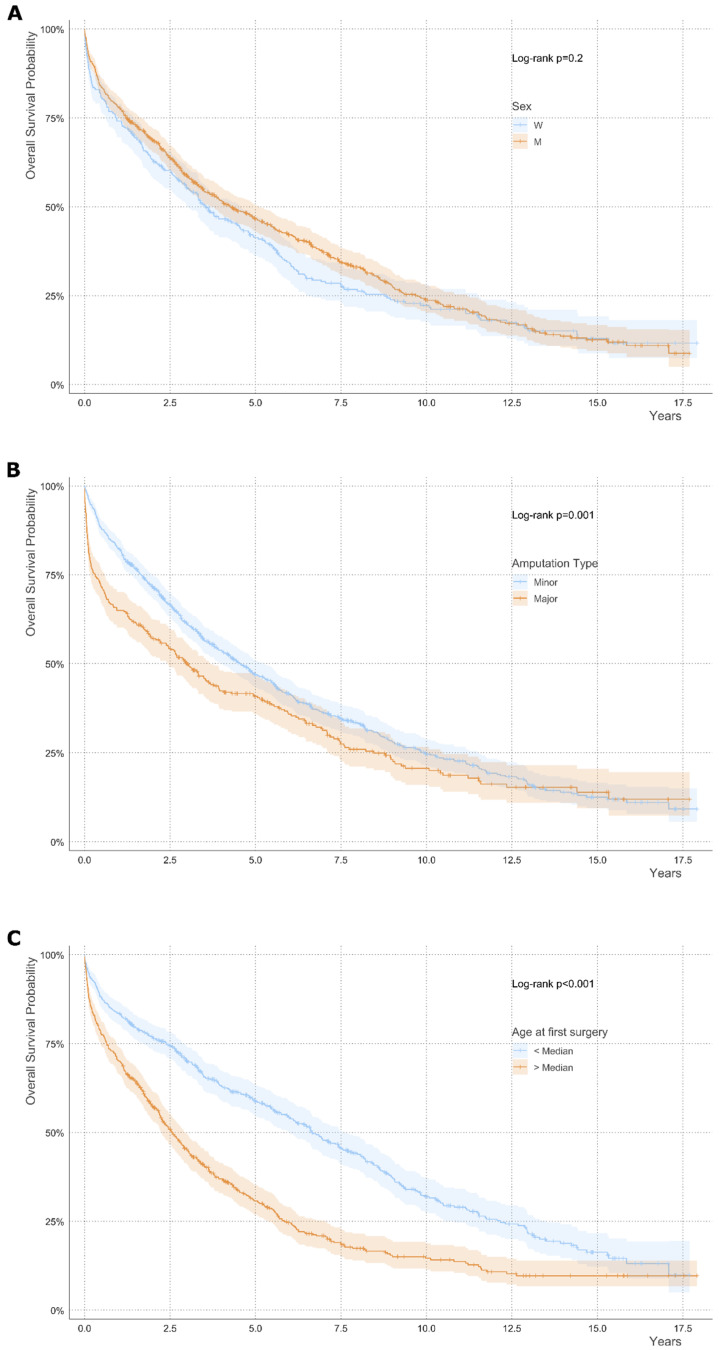
Overall survival probability in bivariate subgroups according to sex, amputation type and age. Subgroup analysis for (**A**) sex, (**B**) amputation type and (**C**) age. Log-rank test *p*-value is indicated for statistical comparison between groups. Shaded areas indicate the 95% confidence intervals. Censored data points are marked by ticks. Univariate analysis is shown.

Expectedly, survival was higher in patients undergoing a minor amputation compared to those with major amputation as a first surgery (Median Survival Time (years): 4.54 [4.03; 5.25] vs. 2.99 [2.44; 3.69], *p* = 0.001 (Log-rank)) (Figure 2).

**Figure 3:**
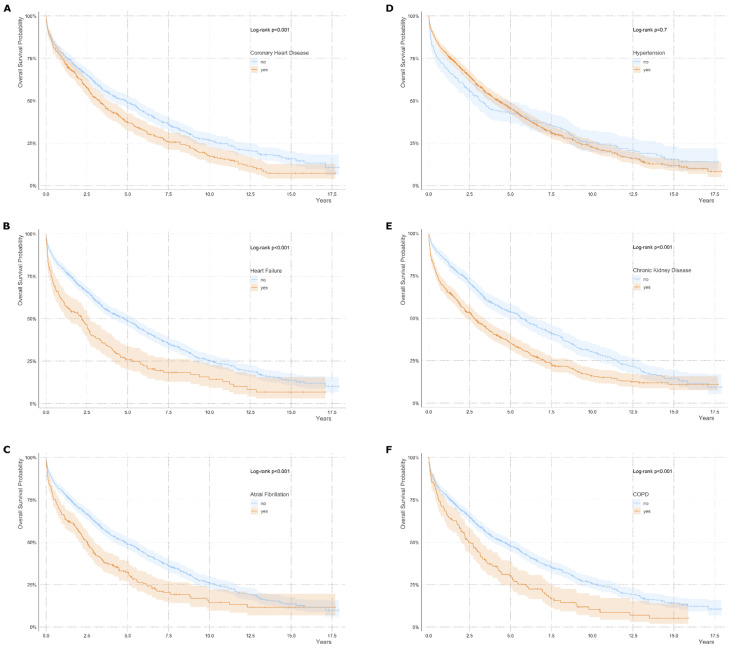
Overall survival probability in bivariate subgroups according to comorbidities. Subgroup analysis for (**A**) coronary heart disease, (**B**) heart failure, (**C**) atrial fibrillation, (**D**) hypertension, (**E**) chronic kidney disease and (**F**) chronic pulmonary disease (COPD) are shown. Diagnoses were based on clinical and laboratory findings. Log-rank test *p*-value is indicated for statistical comparison between groups. Shaded areas indicate the 95% confidence intervals. Censored data points are marked by ticks. COPD, chronic pulmonary disease. Univariate analysis is shown.

The presence of coronary heart disease, heart failure, atrial fibrillation, chronic kidney disease and chronic obstructive pulmonary disease was associated with increased mortality (Figure 3).

Multivariate regression analysis revealed chronic kidney disease, heart failure and major amputation as negative predictors of one-year survival (Supplementary Figure S1). Statin usage was associated with improved overall survival (Supplementary Figure S2).

**Figure 4:**
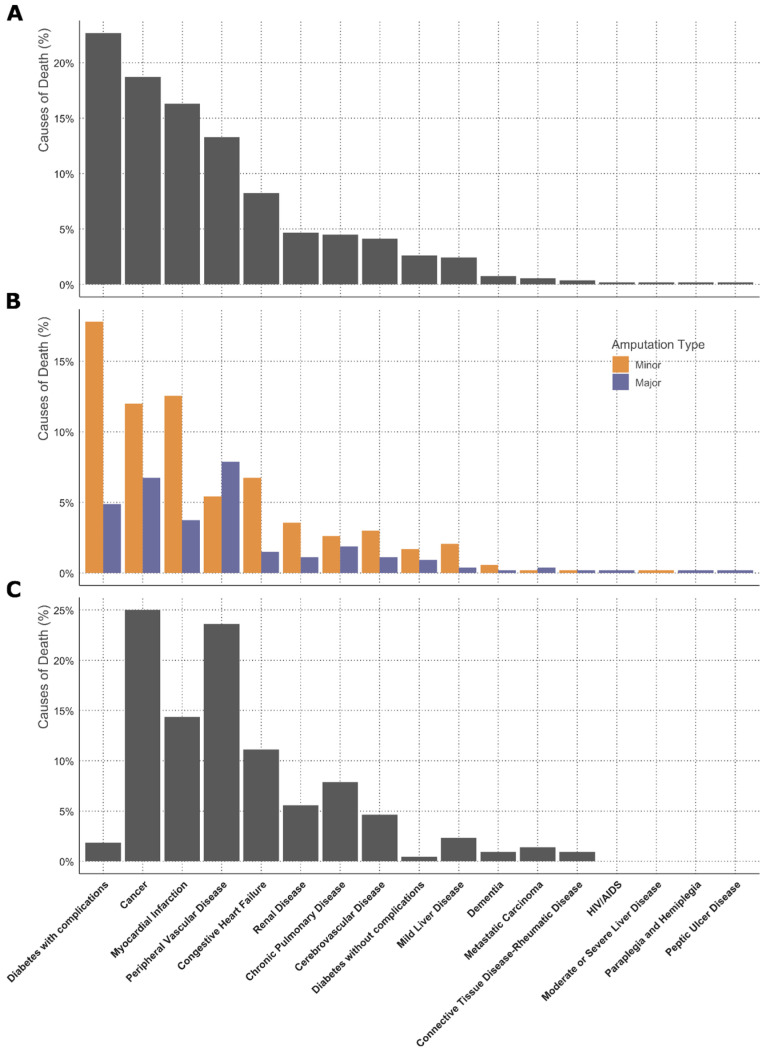
Cause of death according to Charlson comorbidity categories. Causes of death from all patients (**A**), causes of death according to different amputation types at first surgery (**B**) and patients without overt diabetes at time of amputation (**C**), and are shown. Causes of death were based on ICD codes.

Diabetes with complications, cancer, myocardial infarction, peripheral vascular disease and congestive heart failure were the five most frequent causes of death (Figure 4A). Causes of death were significantly different according to the amputation type (Figure 4B, Supplementary Table S1). While in patients whose first surgical procedure was a minor amputation, diabetes, cancer and myocardial infarction were the most frequent causes of death, peripheral artery disease was the most common cause of death in those who initially underwent a major lower-limb amputation (Figure 4B). When excluding patients with overt diabetes at the time of the first amputation, cancer was the most common cause of death followed by peripheral artery disease (Figure 4C). Diabetes, cancer and myocardial infarction were the most common causes of death within one year after the first amputation, while diabetes and peripheral artery disease were the leading causes of death later than one year after the first amputation.

At time of first amputation, female patients were significantly older than males (median age 78.9 vs. 71.4 years) (Table 2). BMI, the frequency of current smokers and prevalence of diabetes were significantly lower in females than in males. In contrast, LDL-C levels were significantly higher in females than in males (median LDL-C 88 mg/dL vs. 77 mg/dL, *p*-value < 0.01). Corresponding to higher LDL-C levels, statin therapy was less common in female than in male patients. Coronary heart disease and chronic pulmonary disease were more common in males than in females. In contrast, chronic kidney disease was more prevalent and more severe in females than in males (51.6% vs. 40.3%, *p* < 0.01). A total of 54.2% of female patients underwent a minor amputation as their first amputation. In males, the rates of minor amputations were significantly higher than in females, comprising 74.1% of first amputations (*p* < 0.01).

### 3.3. Sex-Specific Analysis

Trends in sex-specific characteristics are shown in Table 3 and Table 4.

Remarkably, age at first amputation only significantly increased in men but not in women during the observation period. From P1 to P4 significantly fewer men were active smokers, while rates remained stable in women during this time. Strikingly, and even despite higher baselines levels in P1, LDL-C levels only decreased in male but not in female patients during the observation period. In P4, median LDL-C was 92 mg/dL in females and 70 mg/dL in men. Overall, statin usage increased in both sexes from P1 to P4 but remained almost 20% lower in female patients compared to male patients.

The prevalence of hypertension significantly increased only in men over time, while remaining stable in women. The diagnosis of coronary heart disease and atrial fibrillation significantly increased in men with time and remained stable in women. In females, chronic pulmonary disease increased during the observation period mainly to higher rates in P4; however, the absolute number of affected patients was low, probably overestimating the trend.

Major amputations were performed more frequently in females than in males as first surgical intervention. In females, the cumulative survival probability was higher in patients with minor amputations as a first intervention than in those with major amputations, while the cumulative survival probability was similar in males with minor or major amputations (Supplementary Figure S3). The cumulative survival probability was lowest in females who underwent major amputation as the first surgical intervention. The causes of death significantly differed between females and males (Supplementary Table S1).

**Figure 5:**
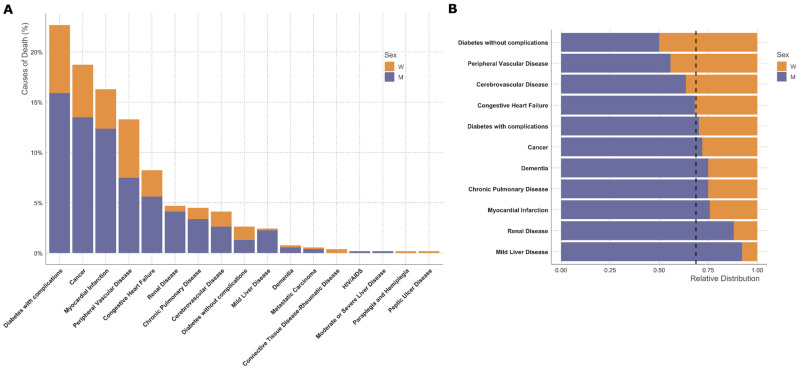
Sex differences in causes of death. Absolute counts of different causes of death are shown in (**A**) and relative distribution of selected Charlson categories in (**B**). Causes of death were based on ICD codes. The dashed line indicates the overall ratio of male to female patients in this study, which is 0.68.

Death due to diabetes without complications, peripheral vascular disease and cerebrovascular disease were more common in females, while death from liver or renal disease were markedly more common in males than in females (Figure 5).

## 4. Discussion

Lower-limb amputation is associated with a dramatic loss in quality of life including mobility, self-esteem and markedly increased mortality [1,32]. Peripheral artery disease and diabetes are the most common causes of non-traumatic lower-limb amputations and, despite several prevention programs and campaigns, the prevalence of peripheral artery disease and type 2 diabetes is still increasing [33,34].

In parallel to this increasing prevalence, novel cardiovascular risk-factor treatments and treatment goals have evolved. In this study, we analyzed whether novel therapies and risk-factor treatment targets are reflected by reduced cardiovascular risk burden and improved outcomes in patients undergoing non-traumatic lower-limb amputation.

Mortality rates were high and remained unchanged from 2006 to 2022, resulting in a median survival time of 4.03 years in patients undergoing non-traumatic lower limb amputation. Our finding is in contrast to very recent data showing a significant reduction in avoidable mortality from cardiovascular disease, including peripheral artery disease in the European Union from 1995 to 2020 [35]. In this study, mortality from peripheral artery disease or atherosclerotic disease other than ischemic heart disease and cerebrovascular disease was considered as avoidable through better prevention and/or early and efficient treatment.

Actually, in our analysis, patients were characterized by a high comorbidity burden Importantly the presence of coronary heart disease, heart failure, atrial fibrillation, chronic kidney disease and chronic pulmonary disease was associated with increased mortality in our study, underlining the close relation between a high comorbidity burden and poor outcome.

The prevalence of coronary heart disease, chronic obstructive pulmonary disease and atrial fibrillation increased significantly during the observation period, which mainly might be due to the temporal trend towards an older age at the time of the first amputation in male patients.

In our study, chronic kidney disease was more frequent and severe in females than in males. These data are consistent with the literature showing a higher prevalence of chronic kidney disease in women [36]. Mechanistically, the natural decline of renal function with age and the underestimation of eGFR determined by the CKD-EPI formula might contribute to this finding [37,38].

Besides comorbidities, older age was also correlated with a poorer outcome in this study. Underlining the risk of older age, Li Q et al. [3] recently reported that mortality was up to 39.2% in patients older than 80 years who had undergone revascularization within 3 months before amputation.

Logistic regression analysis revealed chronic kidney disease, heart failure and performance of major amputations as predictors of 1-year mortality.

Both chronic kidney disease and major amputations were more common in women than in men, further underlining the need for effective screening measures and treatment of cardiovascular risk factors, particularly in women.

The median age at the time of the first amputation increased significantly from 2006 to 2022, with females being markedly older than male patients. The impairment of endothelial dysfunction usually occurs one decade later in women than in males, which might explain the marked sex difference in median age [14,39,40,41]. Noteworthy, age at the time of the first amputation increased only in male but not in female patients in our study, suggesting improvements in management and treatments in males only. On the other hand, male preponderance even increased with time with more than 70% of first amputations being performed in males in P 4. When analyzing the type of first amputation, the rates of major amputations were significantly higher in females than in males which might be explained by lower diabetes rates in females. However, lower screening rates and less aggressive treatment of cardiovascular risk factors in women might also contribute to higher rates of major amputations [42,43]. Strikingly, cumulative survival probability was dependent on amputation type in females but not in males, with the worst outcome for female patients undergoing a major amputation as the first surgical intervention. This might be explained by previous data showing that women are less likely treated with evidence-based medical therapy after endovascular revascularization for peripheral artery disease [44]. Underlining these previous data, statin usage was less common in females than in males in our cohort, further stressing the urgent need to improve medical treatment for cardiovascular risk reduction, particularly in women.

Diabetes-related complications, cancer and myocardial infarction were the most common causes of death in the whole cohort. While diabetes and vascular complications were the most common causes of death in females, males more frequently died from non-cardiovascular diseases such as renal disease, liver disease or chronic pulmonary disease.

This sex-discrepancy with higher mortality from peripheral artery disease, diabetes and cerebrovascular disease in women might be at least partly explained by a worse cardiovascular risk profile and poorer therapeutic management in our study: smoking behavior improved in males but not in females through the study period. Importantly, LDL-C significantly decreased in males, while there was a trend towards higher LDL-C levels in females throughout the study, suggesting clinical inertia or reduced adherence to lipid-lowering therapy in female patients. Indeed, females were treated with statins less commonly than males in our study, and the overall frequency of statin usage was low. Our data go hand in hand with other reports showing better LDL-cholesterol control in male than in female high-risk patients [45,46].

These data underline the need to address and reduce cardiovascular risk, especially in women with lower-limb amputation, particularly as previous data have shown that intensive lipid-lowering treatment reduces major adverse limb events [47]. Improved amputation rates have also been reported for GLP-1 agonist treatment in patients with type 2 diabetes [48].

In contrast to smoking behavior and hypercholesterinemia, the prevalence of hypertension increased in both sexes during the observation period and was equally common in female and male patients [24,49,50]. The increasing rates of hypertension might partially be explained by the recommendation of lowering the treatment threshold in 2007 [51].

## 5. Conclusions

Our data show increased age and an overall improved cardiovascular risk profile in males while no relevant amelioration was found in female patients undergoing lower-limb amputation. Despite novel treatment options and tighter treatment goals for cardiovascular risk factors, mortality remains very high, probably at least partly reflecting older age at the first time of amputation in males and poor cardiovascular risk management in females. Our data indicate that preventive measures and treatment of patients with peripheral artery disease and diabetes need to be intensified, especially in females.

### Study Limitations

According to the retrospective design of the study, a selection bias cannot be ruled out and completeness of the data cannot be guaranteed. Time-restricted limitations in patients’ care during the SARS-CoV-2 pandemic might have led to delayed diagnosis and/or treatments affecting the number and type of amputations. Additionally, post-operative rehabilitation, which was not included in this study, might have influenced the outcome of patients. Another limitation of this study is that data on antidiabetic and antihypertensive medications were not included in this analysis. However, differences in antidiabetic or antihypertensive medication might have influenced the outcome of the studied patients. While data on comorbidities were based on clinical and laboratory findings obtained from medical records, information on causes of death were based on ICD codes.

This study population exclusively encompasses patients who underwent lower-limb amputation at the University Hospital of Vascular Surgery Innsbruck and thus it might underestimate the number of patients with minor amputations as some patients might also be treated in other hospitals. Importantly, the catchment area of the University Hospital has remained unchanged between 2006 and 2022 and covers a population of about 1 million inhabitants in Western Austria. While our study is not suitable for estimating incidences of non-traumatic lower-limb amputations, our data demonstrate high mortality and morbidity in patients with non-traumatic lower-limb amputations with a poorer outcome in women than in men.

## Figures and Tables

**Table 1:** Baseline characteristics and amputation risk factors at first amputation. Numerical data are presented as median with 25th and 75th percentiles in brackets. If not otherwise specified, categorial data are presented as percent of “yes”. For numerical values, Kruskal–Wallis Test was used to compare groups; for categorial variables Chi-squared test was used. Trend testing was performed with Spearman’s rank correlation for numerical data and with Mantel–Haenszel test for categorial data. *p*.overall indicates global statistical testing across the grouping variable, whereas *p*.trend indicates trend testing over time. For trend testing either Spearman’s test (numerical non-normal data) or Mantel–Haenszel test was used (categorical data). Diagnoses were based on clinical and laboratory findings.

	[ALL]	2006–2010	2011–2015	2016–2019	2020–2022	*p*.overall	*p*.trend
	*N* = 1107	*N* = 361	*N* = 290	*N* = 250	*N* = 206		
Age (years)	73.6 [64.7; 81.5]	72.0 [62.7; 80.5]	73.9 [66.0; 82.0]	73.4 [64.1; 81.4]	75.3 [66.4; 82.1]	0.071	0.033
<Median	553 (50.0%)	192 (53.2%)	140 (48.3%)	127 (50.8%)	94 (45.6%)	0.328	0.194
>Median	554 (50.0%)	169 (46.8%)	150 (51.7%)	123 (49.2%)	112 (54.4%)		
Female Sex	345 (31.2%)	135 (37.4%)	92 (31.7%)	59 (23.6%)	59 (28.6%)	0.006	0.007
BMI (kg/m^2^)	24.8 [22.0; 28.5]	25.0 [21.8; 28.0]	24.4 [21.6; 27.9]	26.4 [22.9; 29.6]	24.4 [22.0; 28.2]	0.004	0.471
*Risk factors*							
Smoking	279 (28.0%)	83 (31.7%)	83 (29.5%)	58 (23.2%)	55 (27.0%)	<0.001	0.011
Diabetes	608 (55.0%)	224 (62.2%)	134 (46.2%)	142 (56.8%)	108 (52.4%)	0.001	0.124
LDL-C (mg/dL)	79.0 [59.0; 102]	87.5 [68.0; 117]	77.0 [57.0; 98.0]	78.0 [59.0; 99.0]	74.0 [52.0; 98.5]	0.001	0.003
Hypertension	856 (77.5%)	259 (72.1%)	210 (72.4%)	208 (83.2%)	179 (86.9%)	<0.001	<0.001
Statin usage	563 (50.9%)	124 (34.3%)	158 (54.5%)	159 (63.6%)	122 (59.2%)	<0.001	<0.001
*Comorbidities*							
CHD	401 (36.3%)	123 (34.2%)	81 (27.9%)	102 (40.8%)	95 (46.1%)	0.001	0.003
Heart failure	182 (16.5%)	59 (16.5%)	35 (12.1%)	53 (21.2%)	35 (17.0%)	0.071	0.330
CKD	478 (43.9%)	157 (44.4%)	134 (46.5%)	114 (46.0%)	73 (36.5%)	0.143	0.197
Creatinine (mg/dL)	1.07 [0.81; 1.62]	1.06 [0.80; 1.66]	1.09 [0.82; 1.72]	1.14 [0.86; 1.53]	1.00 [0.77; 1.41]	0.141	0.272
eGFR (mL/min/1.73 m^2^)						0.118	0.068
<30	174 (16.0%)	66 (18.6%)	50 (17.4%)	33 (13.3%)	25 (12.5%)		
30–60	304 (27.9%)	91 (25.7%)	84 (29.2%)	81 (32.7%)	48 (24.0%)		
>60	612 (56.1%)	197 (55.6%)	154 (53.5%)	134 (54.0%)	127 (63.5%)		
Atrial Fibrillation	267 (24.2%)	65 (18.1%)	62 (21.4%)	80 (32.1%)	60 (29.1%)	0.001	<0.001
COPD	177 (16.0%)	45 (12.5%)	45 (15.5%)	46 (18.5%)	41 (20.0%)	0.107	0.020
*Amputation Type*						0.263	0.230
Minor	752 (67.9%)	240 (66.5%)	188 (64.8%)	181 (72.4%)	143 (69.4%)		
Major	355 (32.1%)	121 (33.5%)	102 (35.2%)	69 (27.6%)	63 (30.6%)		

**Table 2:** Sex differences in baseline characteristics. Numerical data are presented as median with 25th and 75th percentiles in brackets. If not otherwise specified, categorial data are presented as percent of “yes”. For numerical values, Kruskal–Wallis Test was used to compare groups; for categorial variables Chi-squared test was used. Diagnoses were based on clinical and laboratory findings.

	All *N* = 1107	Females *N* = 345	Males *N* = 762	*p*-Value
Age (years)	73.6 [64.7; 81.5]	78.9 [70.3; 86.8]	71.4 [63.0; 79.3]	<0.001
<Median	553 (50.0%)	115 (33.3%)	438 (57.5%)	<0.001
>Median	554 (50.0%)	230 (66.7%)	324 (42.5%)	
BMI (kg/m^2^)	24.8 [22.0; 28.5]	23.9 [20.2; 28.6]	25.1 [22.6; 28.5]	0.002
***Risk factors***				
Smoking	279 (28.0%)	65 (21.2%)	214 (31.0%)	0.002
Diabetes	608 (55.0%)	167 (48.4%)	441 (58.0%)	0.005
LDL-C (mg/dL)	79.0 [59.0; 102]	88.0 [63.0; 115]	77.0 [57.0; 98.0]	0.002
Hypertension	856 (77.5%)	266 (77.1%)	590 (77.6%)	0.906
Statin usage	563 (50.9%)	142 (41.2%)	421 (55.2%)	<0.001
***Comorbidities***				
CHD	401 (36.3%)	94 (27.2%)	307 (40.3%)	<0.001
Heart failure	182 (16.5%)	51 (14.8%)	131 (17.3%)	0.399
CKD	478 (43.9%)	176 (51.6%)	302 (40.3%)	0.001
Creatinine (mg/dL)	1.07 [0.81; 1.62]	0.98 [0.73; 1.50]	1.12 [0.85; 1.65]	<0.001
eGFR (mL/min/1.73 m^2^)				0.003
<30	174 (16.0%)	64 (18.8%)	110 (14.7%)	
30–60	304 (27.9%)	112 (32.8%)	192 (25.6%)	
>60	612 (56.1%)	165 (48.4%)	447 (59.7%)	
Atrial Fibrillation	267 (24.2%)	77 (22.3%)	190 (25.0%)	0.399
COPD	177 (16.0%)	36 (10.4%)	141 (18.6%)	0.002
***Amputation Type***				<0.001
Minor	752 (67.9%)	187 (54.2%)	565 (74.1%)	
Major	355 (32.1%)	158 (45.8%)	197 (25.9%)	

**Table 3:** Sex-specific and period-specific characteristics of female patients. Numerical data are presented as median with 25th and 75th percentiles in brackets. If not otherwise specified, categorial data are presented as percent of “yes”. For numerical values, Kruskal–Wallis Test was used to compare groups, for categorial variables Chi-squared test was used. *p*.overall indicates global statistical testing across the grouping variable, whereas *p*.trend indicates trend testing over time. For trend testing either Spearman’s test (numerical non-normal data) or Mantel–Haenszel test was used (categorical data). Diagnoses were based on clinical and laboratory findings.

	[ALL]	2006–2010	2011–2015	2016–2019	2020–2022	*p*.overall	*p*.trend
	*N* = 345	*N* = 135	*N* = 92	*N* = 59	*N* = 59		
Age (years)	78.9 [70.3; 86.8]	79.3 [69.0; 85.5]	80.8 [72.9; 89.3]	77.1 [70.1; 86.5]	78.3 [68.3; 86.9]	0.678	0.893
<Median	115 (33.3%)	46 (34.1%)	27 (29.3%)	22 (37.3%)	20 (33.9%)	0.774	0.893
>Median	230 (66.7%)	89 (65.9%)	65 (70.7%)	37 (62.7%)	39 (66.1%)		
BMI (kg/m^2^)	23.9 [20.2; 28.6]	23.4 [19.8; 26.9]	24.4 [19.3; 28.7]	26.0 [21.2; 30.0]	23.2 [21.7; 26.6]	0.459	0.423
***Risk factors***							
Smoking	65 (21.2%)	24 (24.0%)	19 (21.3%)	8 (13.6%)	14 (24.1%)	0.090	0.423
Diabetes	167 (48.4%)	70 (51.9%)	41 (44.6%)	30 (50.8%)	26 (44.1%)	0.710	0.671
LDL-C (mg/dL)	88.0 [63.0; 115]	88.5 [61.8; 124]	77.0 [63.0; 100]	94.0 [70.0; 110]	92.0 [57.0; 116]	0.658	0.893
Hypertension	266 (77.1%)	99 (73.3%)	63 (68.5%)	53 (89.8%)	51 (86.4%)	0.032	0.052
Statin usage	142 (41.2%)	37 (27.4%)	42 (45.7%)	36 (61.0%)	27 (45.8%)	0.001	0.007
***Comorbidities***							
CHD	94 (27.2%)	42 (31.1%)	18 (19.6%)	15 (25.4%)	19 (32.2%)	0.468	0.962
Heart Failure	51 (14.8%)	21 (15.6%)	12 (13.2%)	11 (18.6%)	7 (11.9%)	0.767	0.893
CKD	176 (51.6%)	71 (53.4%)	47 (51.6%)	33 (55.9%)	25 (43.1%)	0.678	0.613
Creatinine (mg/dL)	0.98 [0.73; 1.50]	1.01 [0.74; 1.63]	0.98 [0.71; 1.41]	1.01 [0.78; 1.21]	0.84 [0.69; 1.41]	0.678	0.423
eGFR (mL/min/1.73 m^2^)						0.459	0.423
<30	64 (18.8%)	30 (22.6%)	17 (18.7%)	6 (10.2%)	11 (19.0%)		
30–60	112 (32.8%)	41 (30.8%)	30 (33.0%)	27 (45.8%)	14 (24.1%)		
>60	165 (48.4%)	62 (46.6%)	44 (48.4%)	26 (44.1%)	33 (56.9%)		
Atrial Fibrillation	77 (22.3%)	26 (19.3%)	18 (19.6%)	17 (28.8%)	16 (27.1%)	0.606	0.423
COPD	36 (10.4%)	10 (7.41%)	10 (10.9%)	5 (8.47%)	11 (18.6%)	0.459	0.243
***Amputation Type***						0.476	0.701
Minor	187 (54.2%)	71 (52.6%)	46 (50.0%)	39 (66.1%)	31 (52.5%)		
Major	158 (45.8%)	64 (47.4%)	46 (50.0%)	20 (33.9%)	28 (47.5%)		

**Table 4:** Sex-specific and period-specific characteristics of male patients. Numerical data are presented as median with 25th and 75th percentiles in brackets. If not otherwise specified, categorial data are presented as percent of “yes”. For numerical values, Kruskal–Wallis Test was used to compare groups; for categorial variables Chi-squared test was used. *p*.overall indicates global statistical testing across the grouping variable, whereas *p*.trend indicates trend testing over time. For trend testing either Spearman’s test (numerical non-normal data) or Mantel–Haenszel test was used (categorical data). Diagnoses were based on clinical and laboratory findings.

	[ALL]	2006–2010	2011–2015	2016–2019	2020–2022	*p*.overall	*p*.trend
	*N* = 762	*N* = 226	*N* = 198	*N* = 191	*N* = 147		
Age (years)	71.4 [63.0; 79.3]	68.1 [60.1; 76.6]	71.7 [64.5; 77.9]	72.3 [63.0; 80.7]	73.5 [66.0; 81.2]	0.001	<0.001
<Median	438 (57.5%)	146 (64.6%)	113 (57.1%)	105 (55.0%)	74 (50.3%)	0.065	0.010
>Median	324 (42.5%)	80 (35.4%)	85 (42.9%)	86 (45.0%)	73 (49.7%)		
BMI (kg/m^2^)	25.1 [22.6; 28.5]	25.6 [23.1; 28.6]	24.4 [21.8; 27.7]	26.6 [23.1; 28.9]	24.5 [22.3; 29.0]	0.007	0.779
***Risk factors***							
Smoking	214 (31.0%)	59 (36.4%)	64 (33.3%)	50 (26.2%)	41 (28.1%)	0.001	0.010
Diabetes	441 (58.0%)	154 (68.4%)	93 (47.0%)	112 (58.6%)	82 (55.8%)	0.001	0.098
LDL-C (mg/dL)	77.0 [57.0; 98.0]	87.5 [68.0; 107]	76.5 [53.8; 98.0]	75.0 [56.8; 95.5]	70.0 [52.0; 89.0]	0.001	<0.001
Hypertension	590 (77.6%)	160 (71.4%)	147 (74.2%)	155 (81.2%)	128 (87.1%)	0.003	<0.001
Statin usage	421 (55.2%)	87 (38.5%)	116 (58.6%)	123 (64.4%)	95 (64.6%)	<0.001	<0.001
***Comorbidities***							
CHD	307 (40.3%)	81 (36.0%)	63 (31.8%)	87 (45.5%)	76 (51.7%)	0.001	0.001
Heart failure	131 (17.3%)	38 (17.0%)	23 (11.6%)	42 (22.0%)	28 (19.0%)	0.075	0.261
CKD	302 (40.3%)	86 (38.9%)	87 (44.2%)	81 (42.9%)	48 (33.8%)	0.272	0.537
Creatinine (mg/dL)	1.12 [0.85; 1.65]	1.10 [0.82; 1.71]	1.15 [0.88; 1.87]	1.19 [0.87; 1.59]	1.04 [0.81; 1.40]	0.110	0.365
eGFR (mL/min/1.73 m^2^)						0.385	0.261
<30	110 (14.7%)	36 (16.3%)	33 (16.8%)	27 (14.3%)	14 (9.86%)		
30–60	192 (25.6%)	50 (22.6%)	54 (27.4%)	54 (28.6%)	34 (23.9%)		
>60	447 (59.7%)	135 (61.1%)	110 (55.8%)	108 (57.1%)	94 (66.2%)		
Atrial Fibrillation	190 (25.0%)	39 (17.3%)	44 (22.2%)	63 (33.2%)	44 (29.9%)	0.002	0.001
COPD	141 (18.6%)	35 (15.6%)	35 (17.7%)	41 (21.6%)	30 (20.5%)	0.423	0.193
***Amputation Type***						0.806	0.756
Minor	565 (74.1%)	169 (74.8%)	142 (71.7%)	142 (74.3%)	112 (76.2%)		
Major	197 (25.9%)	57 (25.2%)	56 (28.3%)	49 (25.7%)	35 (23.8%)		

## Data Availability

All data supporting the findings of this study are available within the paper and its Supplementary Information.

## Supplementary Materials

The following supporting information can be downloaded at: https://www.mdpi.com/article/10.3390/jcm14124030/s1, Table S1: Sex-specific causes of death according to Charlson comorbidity categories in total numbers and percentage of decedents. Causes of death were based on ICD codes; Figure S1: Logistic regression analysis using clinically relevant covariates for survival data 12 months after initial surgery. OR, Odds Ratio; Figure S2: Overall survival probability in bivariate subgroups according to statin use. Log-rank test *p*-value is indicated for statistical comparison between groups. Shaded areas indicate the 95% confidence intervals. Censored data points are marked by ticks. Univariate analysis is shown; Figure S3: Cumulative survival probability in (a) female and (b) male patients according to the amputation type at time of first surgical intervention.
