# Supplementary material for: Non-Traumatic Lower-Limb Amputations: Outcome, Sex-Differences, Comorbidity Patterns and Temporal Trends from 2006 to 2022
Source: J Clin Med. 2025 Jun 6;14(12):4030. doi: 10.3390/jcm14124030 (PMC12194082; doi:10.3390/jcm14124030)
Supplement: Supplementary file 1 [file jcm-14-04030-s001.zip › jcm-3653060-supplementary/Supplement Table S1.pdf]

Supplement Table S1

	Sex				Amputation Type		
	**[ALL]**	**Female**	**Male**	**p.overall**	**Minor**	**Major**	**p.overall**
	N=216	***N=73***	***N=143***		***N=119***	***N=97***	
Cause of Death:				0.014			0.005
Cancer	54 (25.0%)	13 (17.8%)	41 (28.7%)		29 (24.4%)	25 (25.8%)	
Cerebrovascular Disease	10 (4.63%)	5 (6.85%)	5 (3.50%)		8 (6.72%)	2 (2.06%)	
Chronic Pulmonary Disease	17 (7.87%)	5 (6.85%)	12 (8.39%)		7 (5.88%)	10 (10.3%)	
Congestive Heart Failure	24 (11.1%)	10 (13.7%)	14 (9.79%)		19 (16.0%)	5 (5.15%)	
Connective Tissue Disease-Rheumatic Disease	2 (0.93%)	2 (2.74%)	0 (0.00%)		1 (0.84%)	1 (1.03%)	
Dementia	2 (0.93%)	1 (1.37%)	1 (0.70%)		1 (0.84%)	1 (1.03%)	

	Sex				Amputation Type		
	[ALL]	Female	Male	p.overall	Minor	Major	p.overall
	N=216	*N=73*	*N=143*		*N=119*	*N=97*	
Diabetes with complications	4 (1.85%)	2 (2.74%)	2 (1.40%)		0 (0.00%)	4 (4.12%)	
Diabetes without complications	1 (0.46%)	1 (1.37%)	0 (0.00%)		0 (0.00%)	1 (1.03%)	
Metastatic Carcinoma	3 (1.39%)	1 (1.37%)	2 (1.40%)		1 (0.84%)	2 (2.06%)	
Mild Liver Disease	5 (2.31%)	0 (0.00%)	5 (3.50%)		3 (2.52%)	2 (2.06%)	
Myocardial Infarction	31 (14.4%)	6 (8.22%)	25 (17.5%)		22 (18.5%)	9 (9.28%)	
Peripheral Vascular Disease	51 (23.6%)	25 (34.2%)	26 (18.2%)		20 (16.8%)	31 (32.0%)	
Renal Disease	12 (5.56%)	2 (2.74%)	10 (6.99%)		8 (6.72%)	4 (4.12%)	
