# Supplementary figures and images for: Non-Traumatic Lower-Limb Amputations: Outcome, Sex-Differences, Comorbidity Patterns and Temporal Trends from 2006 to 2022
Source: J Clin Med. 2025 Jun 6;14(12):4030. doi: 10.3390/jcm14124030 (PMC12194082; doi:10.3390/jcm14124030)

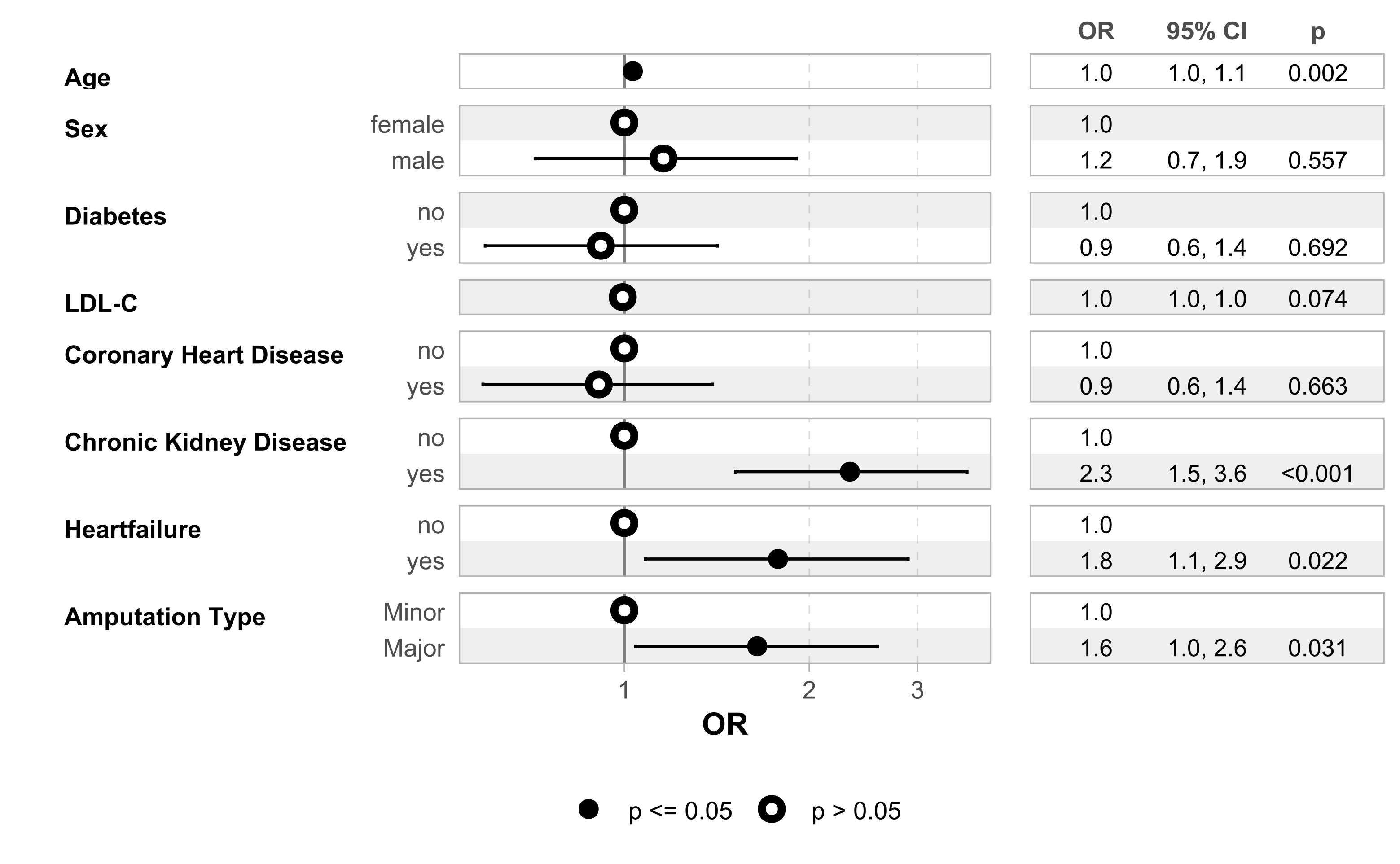

Supplement: Supplementary file 1 [file jcm-14-04030-s001.zip › jcm-3653060-supplementary/Supplementary Figure S1.tif]

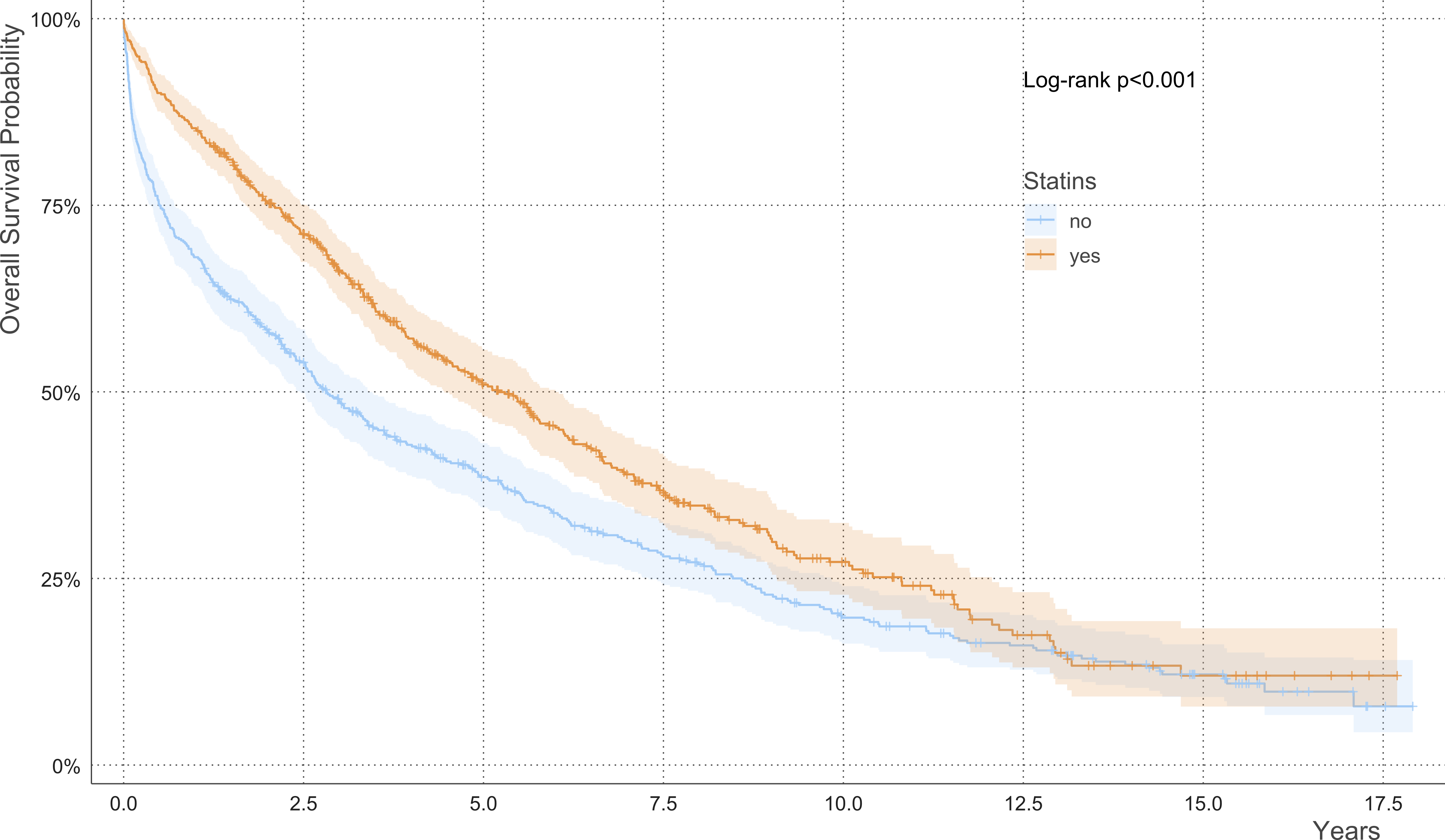

Supplement: Supplementary file 1 [file jcm-14-04030-s001.zip › jcm-3653060-supplementary/Supplementary Figure S2.tif]

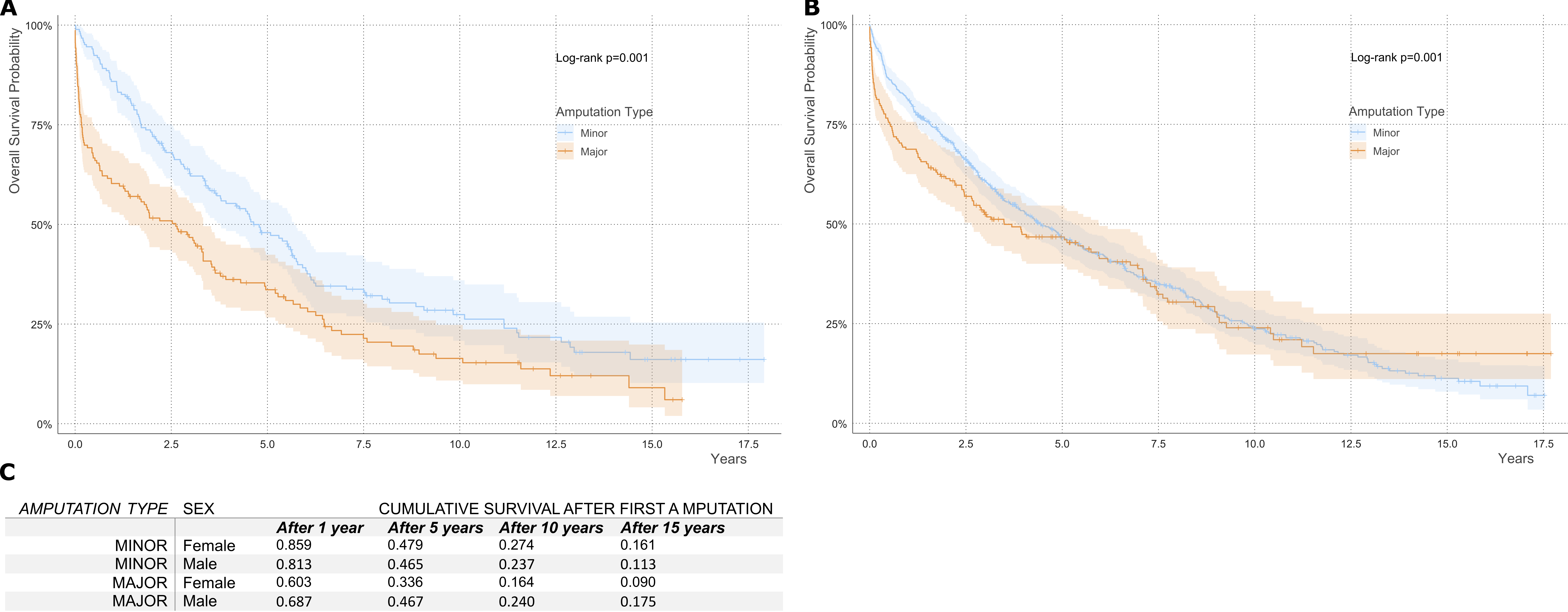

Supplement: Supplementary file 1 [file jcm-14-04030-s001.zip › jcm-3653060-supplementary/Supplementary Figure S3.tif]
